# The role of brand commitment and external information in urban consumers’ organic produce choices: Evidence from Japan and China

**DOI:** 10.1371/journal.pone.0337225

**Published:** 2025-11-21

**Authors:** Runan Yang, Hironori Yagi, Katsuhito Fuyuki

**Affiliations:** 1 Faculty of Agriculture, Ibaraki University, Ibaraki, Japan; 2 Graduate School of Agricultural and Life Sciences, The University of Tokyo, Tokyo, Japan; 3 Graduate School of Agricultural Sciences, Tohoku University, Sendai, Japan; Università degli Studi di Milano: Universita degli Studi di Milano, ITALY

## Abstract

Although organic agriculture brands are growing globally, urban consumer uptake in East Asia remains modest. We examine how brand commitment and external certification information shape willingness to pay (WTP) for organic vegetables based on multiple-store memory model. Online surveys of adult urban consumers in Japan (n = 412) and China (n = 422) are followed by a choice experiment in which respondents are randomly assigned to external certification information. Using latent profile analysis, we segment consumers’ brand commitment into five groups and estimate conditional logit models of choice and WTP. Results show that higher brand commitment and external certification information increase WTP for organic cabbage and tomatoes. Among Chinese consumers, information does not significantly affect WTP for carrots, indicating product-specific value perceptions. In Japan, information about semi-organic certification raises WTP for tomatoes, especially among high-brand commitment segments. The study contributes theoretically by linking brand commitment with information sensitivity to explain consumer heterogeneity, and by highlighting the importance of tailored marketing strategies. Our findings emphasize the need for targeted communication to enhance organic produce consumption in urban Asian markets.

## Introduction

Amid a global shift towards environmental sustainability and health-conscious lifestyles, the area occupied by organic farming in the EU increased by 56% from 2012 to 2020 [1,2]. Yet, in East Asia, particularly in the urban areas of China and Japan, the adoption of organic food consumption has lagged global trends. A primary challenge is consumers’ limited awareness and understanding of organic produce [3–5], emphasizing the need for enhanced consumer knowledge to drive market expansion.

While organic vegetables may not carry pronounced corporate branding like processed foods, the organic certification itself acts as a powerful brand marker [6]. This collective “organic” branding becomes the primary driver of consumers’ trust and purchase decisions. However, branding for fresh produce is often subdued due to the inherent nature of these products as agricultural commodities [7,8]. Moreover, in markets with low organic awareness, external information (EI) becomes pivotal [9–11]. More importantly, many studies point out that external information can shape purchase intentions, making it a critical tool for market expansion [12–14]. Such research has suggested branding and information strategies for high-value-added organic produce; comprehensive examination linking WTP with brand is limited [8,13,15–17]. Research has often studied BC and EI in isolation, lacking a unified theoretical framework to explain their interplay.

To address this gap, this study draws on Bettman’s [18] influential multiple-store memory model of consumer choice. Within this framework, we conceptualize brand commitment (BC) as a stable, organized knowledge structure residing in a consumer’s long-term memory (LTM), built from past experiences, trust, and accumulated knowledge. We treat external information as a situational stimulus that enters the short-term memory (STM) to influence immediate decision-making. We refer to consumer engagement with organic produce brands as brand commitment, a construct that shapes consumers’ economic value perception. Furthermore, consumers are constantly exposed to external information, such as labels and definitions, which can influence their choices. While both China and Japan have developed organic certification systems, differences in market maturity and consumer trust suggest that the interplay between LTM and STM may manifest differently, making a cross-national comparison particularly insightful.

To operationalize this theoretical framework, our study advances prior research (e.g., [19]) by integrating two methodological approaches. First, we employ latent profile analysis (LPA) to empirically identify distinct consumer segments based on their brand commitment. Second, we use a choice experiment (CE) to assess how a controlled external information stimulus influences willingness-to-pay across these segments. This integrated approach allows for a theoretically grounded and nuanced analysis of consumer behavior.

This study aims to develop and test a unified model of organic produce choice grounded in consumer information processing theory. Specifically, (1) To segment urban consumers in Japan and China based on their brand commitment; (2) To investigate how these distinct segments influence willingness-to-pay for organic and semi-organic produce; and (3) To examine the effect of external information on willingness-to-pay and explore how this effect differs across consumer segments and national contexts. To achieve these aims, we test the following hypotheses:

- H1a: High BC consumers have a higher WTP for organic produce.
- H1b: High BC consumers have a higher WTP for semi-organic produce.
- H2a: Consumers exposed to EI about organic produce increase WTP for organic produce.
- H2b: Consumers exposed to EI about semi-organic produce increase WTP for semi-organic produce.

## Background and literature review

### Classification of organic produce in China and Japan

**Fig 1:**
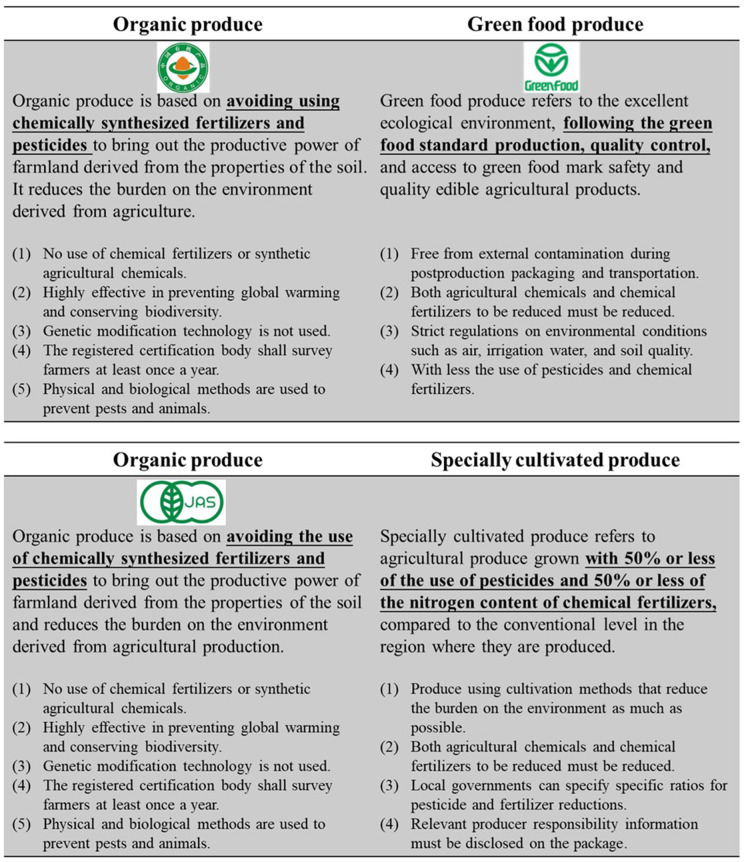
Types of organic produce in Japan and China. Source: CGFDC. http://www.greenfood.agri.cn/. MAFF. https://www.maff.go.jp/j/seisan/kankyo/yuuki/.

Organic agriculture is defined by the International Federation of Organic Agriculture Movements [2] as a production system that sustains the health of soils, ecosystems, and people. Both China and Japan have enacted certification systems for “green food” and “specially cultivated” produce, respectively [3,20]. These categories can be considered “semi-organic”, as their certification requirements are less stringent than those for fully organic farming. Fig 1 shows the category of definitions and labels available in these markets. Oversight for the certification of organic food in China is provided by the China Green Food Development Center (CGFDC), while in Japan it is provided by the Ministry of Agriculture, Forestry and Fisheries (MAFF). Given the high value-added organic produce, consumption in both countries is concentrated in urban areas with higher average incomes [3].

### Organic produce and WTP

The organic market, with its promises of healthier consumption and increased consumer trust, positions itself as a modern, upscale lifestyle choice [21–24]. While studies have delved into the motivations behind consumers’ purchases of organic products, higher prices remain a consistent deterrent [25–28]. In the Asian context, particularly in China and Japan, health, trust, safety [21,29–32], and pollution awareness [33,34] consistently emerge as determinants of WTP for organic produce. For the elderly, health concerns often supersede those of food safety [35]. Additionally, the discrete choice model is often used to analyze consumers’ WTP, suggesting that the high price of organic food significantly influences commodity attribute preferences [14,36,37]. Other determinants include the organic certification system of the Japanese Agricultural Standards [38] and the origin of such produce [39]. However, literature mostly identifies correlates without explaining the cognitive mechanisms underlying response heterogeneity. To explain why some consumers pay a premium while others do not, a theoretical framework that links long-term memory with short-term memory is needed.

### Theoretical framework: The consumer multiple-store memory model

To build a unified model, this study adopts the consumer information processing framework, specifically Bettman’s [18] multiple-store memory model. Rooted in cognitive psychology, this model posits that consumers process information through a series of interacting memory systems: a sensory store, a short-term memory (STM), and a long-term memory (LTM) [18]. External information from the environment first enters the sensory store, and if attended to, is transferred to short-term memory. Short-term memory has limited capacity and duration and supports active processing for immediate decision-making [40]. LTM, in contrast, is a vast and permanent repository of knowledge, experiences, and beliefs, organized into associative networks or schemas [41].

#### Brand commitment.

We conceptualize brand commitment as a complex cognitive schema residing in a consumer’s long-term memory. From a consumer’s perspective, brand commitment denotes psychological attachment to a brand, which drives loyalty and goal pursuit [42,43] and shapes behavior in food purchasing [42,44,45].

While branding is foundational to marketing strategies of globally recognized food brands like Coca-Cola [46], it differs for agricultural produce. Although they may lack the hallmark branding of corporate entities, research suggests that organic certification is a key determinant of how organic produce is treated as a brand [6].

Factors reinforcing brand commitment include trust [43,47], consistent purchasing experiences [48,49], and self-identification with the brand. Informed consumers more often make environmentally sustainable choices [50]. Food taste [51] and sensitivity to food-related risks [52] are associated with higher WTP for organic produce. Intrinsic green consumption and environmental values shape intentions to buy organic foods [21,53]. Demographics such as age, income, and education also influence brand commitment when purchasing organic produce [34,50–52,54,55].

Also, LTM refers to information that consumers have previously received and comprehended in bolstering consumers’ preferences [42,47,49,56,57]. Information flows—peer word-of-mouth, online shopping familiarity, retail availability, and advertising exposure—shape these long-term memory structures [8,15,19,58–60]. With the rise of the internet, online platforms have become pivotal in shaping behavior by enhancing brand commitment [61–63]. Elements such as certification labels, origin, and online media advertising further amplify brand commitment [62,64–67].

Therefore, we employ seven dimensions for organic produce to validate consumers’ brand commitment. We characterize consumers’ brand commitment through three consumer awareness channels: buying experience, knowledge, and trust; and 4 information channels: stores, friends and family, online shopping, and media.

#### External information.

In contrast to the stable structures in long-term memory, we conceptualize external information (EI) as a transient input processed in short-term memory. External information primarily derived from labels and origin descriptions [68–72], enhances consumers’ comprehension of products. By providing concise information, external information acts as a salient “information chunk” in short-term memory. For consumers without well-developed long-term memory scheme for organic produce, this new information can be highly influential.

As many consumers remain unaware of the high value-added attributes of eco-friendly agricultural products, expert knowledge and clear certification definitions serve as crucial tools for shifting purchase intentions [73,74]. In studies related to food choices in Japan, providing information about organic certification can diminish consumers’ perception of flavor [75]. In China, consumers with high trust in organic products show greater purchase intentions than those influenced by external information [76].

These studies demonstrate the impact of external information exchange on consumer preferences for organic products. However, they have not compared preferences for major organic vegetables before and after information exchange in different countries. By failing to account for brand commitment and external information interaction, prior work has not fully captured the dynamic interplay between what consumers already know and what they are told.

## Methodology

### Latent profile analysis

To classify distinct consumer groups influenced by various factors more effectively, scholars use either Latent profile analysis or latent class analysis to classify distinct consumer groups [50,51,77]. Latent profile analysis is a statistical technique that identifies latent characteristics within a sample by utilizing continuous indicators and assuming that these indicators are consistent within each latent class or profile [78–81]. Latent profile analysis postulates the existence of population heterogeneity. This heterogeneity can be sub-divided into observed and unobserved components [82].

Assuming that the continuous indicators follow a normal distribution within each latent class, the latent profile model exemplifies the distribution of the observed scores on a set of continuous indicators $x_{ik}$ (i= 1, …, I) collected from brand commitment. This is modeled as a function of the probability of an individual being a member of a particular latent class ($\pi_{k}$; k = 1, …, K) and the class-specific normal density $f_{k}\left(x_{ik}|\theta_{k}\right)$ as follows [80]:


$$f\left(x_{ik}|\theta \right)=\sum_{k=\text{1}}^{K}\pi_{k}f_{k}\left(x_{ik}|\theta_{k}\right)$$ (1)

### Choice experiments

Choice experiments (CEs) grounded in random utility theory, simulate the purchase of multiple attributes goods to jointly analyze choosers preferences for specific attributes [83,84]. The advantage of CEs lies in the flexibility to design the required attributes, allowing for the combination of different attribute settings to define product characteristics. This enables more detailed analysis of price or attribute preferences through the establishment of interaction terms [85,86]. In this study, we used an unlabeled CEs design created using the mix-and-match method to evaluate the CEs using the R package supportCEs [87].

Conditional logistic (clogit) regression analyzes decision-makers’ choices when they are presented with multiple alternatives with specific attributes. It is commonly applied to discrete choices characterized by impersonal attributes [88]. Individual choices in the presence of heterogeneity can be evaluated using clogit based on the independence of irrelevant alternatives (IIA) condition [89,90]. Therefore, after conducting the control grouped experiments within the CEs, it is highly practical to analyze the data using clogit. The utility function for clogit regression is:


$$U_{nj}=V_{nj}+u_{nj}={\beta x}_{nj}+u_{nj},$$ (2)

where j is a chosen produce attribute, $V_{nj}$ is the observable utility, and $u_{nj}$ is the unobservable error term. $x_{nj}$ is the scenario characteristic (price and organic produce) of the random variable that consumer n should choose.

In Equation (3), for a given consumer n, the choice set j is selected when $U_{nj}$ satisfies the maximum utility of the probability among all available choices. *C* is the set of all alternatives. The probability distribution function for a consumer n to choose under the fixed scenario j option during the observation is


$$P_{nj}=P\left\{V_{nj}+u_{nj}>V_{ni}+u_{ni};j\in C(i\neq j)\right\}=\frac{exp\left(\beta x_{nj}\right)}{\sum_{i=\text{1}}\;exp\left(\beta x_{ni}\right)},$$ (3)

The dummy variable selection set and latent segments are contained in the explanatory variable $x_{nj}$ of the clogit regression. Through latent profile analysis, we identified *k* distinct consumer latent segments. Each segment is represented by a dummy variable $D_{nk}$. To examine how consumers from different segments respond to produce attributes, we introduce an interaction term $D_{nk}{\times Attributes}_{nj}$. Equation (4) illustrates the regression formulation in CEs based on Aizaki and Nishimura [91].


$$U_{nj}=\alpha_{ASC}{ASC}_{n}+{\beta_{P}P}_{nj}+{\beta_{ATT}Attributes}_{nj}+{\beta_{D}D}_{nk}{\times Attributes}_{nj}+u_{nj},$$ (4)

where ${Attributes}_{nj}$ means two produce attribute dummy variables: $\;{ORGA}_{nj}$ and ${SEMIORGA}_{nj}$. ORGA refers to organic produce, SEMIORGA refers to specially cultivated, and green food. Conventional produce was chosen as the baseline group. $\beta_{P}$ is the price parameter. The alternative specific constant (ASC) represents the average of variables that have not been measured [92] and “No choice” were set as 1 and 0, respectively.

Marginal willingness to pay (MWTP) represents the price the customers are MWTP for a particular product feature. It consists of the negative ratio of the parameter $\beta_{ATT}\;$to the price parameter $\beta_{P}$. Equation (5) calculates the MWTP as follows:


$$MWTP=-\frac{\beta_{ATT}}{\beta_{P}},$$ (5)

## Research design and measurement

### Experimental design

The experiment comprised two attributes: price and category, as shown in Table 1.

**Table 1:** Attributes of CEs design.

Attributes		Level-Japan	Level-China
Category		Organic, Semi-organic, Regular	Organic, Semi-organic, Regular
Price (Yen/Yuan)	Cabbage	140, 170, 200, 230, 260	2, 6, 10, 14, 18
	Tomato	170, 210, 250, 290, 330	3, 7, 11, 15, 19
	Carrot	200, 250, 300, 350, 400	2, 6, 10, 14, 18

We selected three representative vegetables for this study—cabbage, tomatoes, and carrots—all widely produced and sold in both countries. These vegetables are commonly available in both organic and conventional forms, helping to minimize heterogeneity in consumer perceptions. In Japan, they are designated items with stabilized prices and high or increasing consumption levels. The selection was further influenced by outlet and production data availability in both countries [20,93]. To prevent cognitive confusion, the vegetables belong to different species (large leafy, tomato, and root vegetables). They are also prepared in similar ways in both countries and consumed either raw or cooked. The prices were sourced from major online supermarket platforms in China and Japan, such as Seiyu, Aeon, Taobao, and JD.com. Prices were divided into five levels, categories divided into three levels: organic, semi-organic, and regular. Offline market surveys were conducted to verify the prices during the corresponding period.

**Fig 2:**
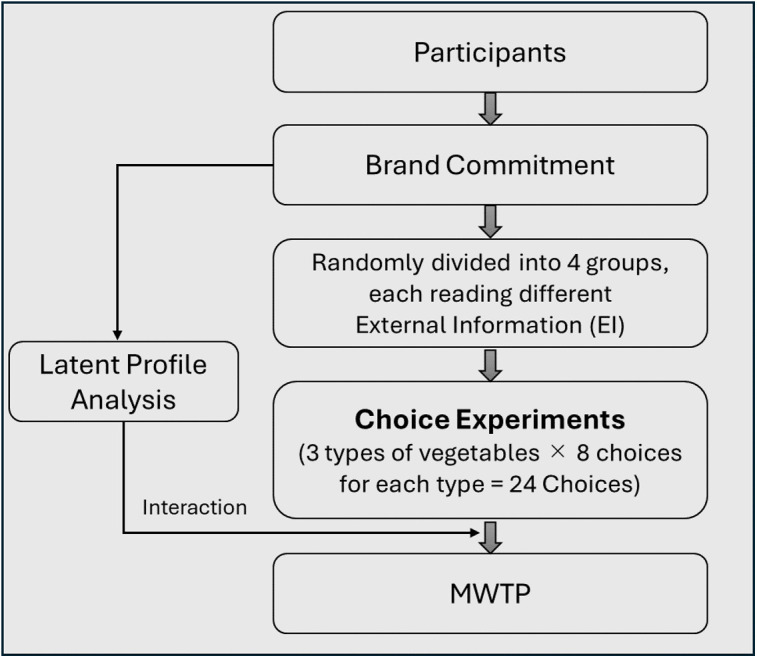
The flow of the choice experiments.

To examine the effect of EI, the flowchart of the CEs is shown in Fig 2.

Participants were randomly assigned to four groups through an online questionnaire, as shown in Table 2. The control group received no external information, while others were presented with definitions of organic produce. For example, the EI-O group read a definition of organic produce (Fig 1 shows the specific information given to each group).

**Table 2:** Reading contents of the external information by group.

Group Name	Read Contents in Japan and China
EI-Control	None (Control group)
EI-O	Organic definition
EI-S	Semi-organic definition
EI-OS	Organic and semi-organic definition

Note: Refer to Fig 1 for the specific contents read by the Chinese and Japanese respondents^*^.

**Fig 3:**
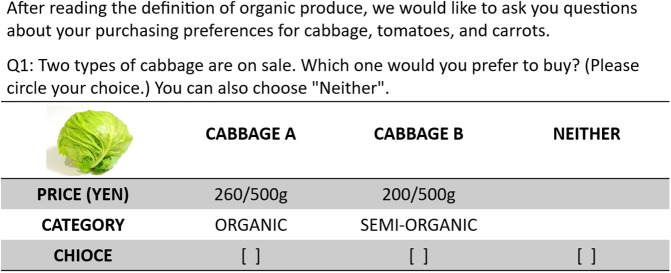
Example of the cards for the choice experiment.

Fig 3 depicts an example of the cards used for the CEs on the Japanese side under the group name “EI-O.” Each vegetable was presented with eight choice sets of varying attributes for respondents to evaluate and choose from. Including the “None” option, each choice set comprised three tasks. Respondents were asked to choose in the order of cabbage-tomato-carrot. There were twenty-four choice sets across the three vegetables. Respondents did not choose their preferred vegetable but rather made choices based on the attributes presented on the vegetable cards. This methodology facilitated the examination of the influence of external information on consumer preferences for the selected vegetables.

The power analysis of sample size calculation methods suggested by Rose and Bliemer [94] and Orme [95] was used to determine the appropriate sample size for our CEs, which consisted of two attributes and up to five levels. Consequently, a sample size of over 100 people per group was considered sufficient.

### Data collection and measurement

We conducted an online survey to collect data on individual characteristics and brand commitment, as detailed in Table 1. To ensure safety during the COVID-19 pandemic, we adopted an online survey method in collaboration with ASMARQ (Japan) and Wenjuanxing (China) for distribution. The shift to online fresh produce markets during this period further supported our choice of method [96]. The questionnaire was translated and verified by scholars and teaching assistants for accuracy, with pre-testing conducted among native-speaking students for clarity. We targeted 20–80-year-old respondents in metropolitan areas who purchased fresh vegetables monthly, reflecting higher organic food consumption in urban settings [25,26,28]. The criteria for selecting respondents residing in government-designated metropolitan cities are provided in S1 Appendix in S1 File.

Participation was entirely anonymous; no names, contact details, IP addresses or other identifying information were recorded. All respondents self-reported an age of 18 years or older, so no minors were involved. Prior to the first question, participants viewed an electronic information sheet describing the study purpose, data-handling procedures, and their right to withdraw. Only those who ticked an “I agree” checkbox could proceed, and the survey platform automatically logged a time stamp as evidence of informed, electronic written consent. Under the University of Tokyo’s *Guidelines for Social and Behavioral Research, anonymous online surveys that are non-medical and do not address psychological issues are exempt from formal Institutional Review Board (IRB) review; therefore, no additional ethics approval was required.

In Japan, 1,097 responses were collected between August 31 and September 2, 2020, with 412 metropolitan-based responses selected from 890 valid submissions (81.13% validity rate). In China, 886 responses were collected between September 22 and 23, 2021, with 422 metropolitan-based responses selected from 652 valid submissions (73.59% validity rate). Chi-square and proportion tests confirmed sample representativeness against the 2020 Population Census and the 2022 Comprehensive Survey of Living Conditions. Invalid responses were excluded. Table 3 outlines the demographic breakdown of the two samples, highlighting key differences such as age distribution and the presence of children under 18.

**Table 3:** Distribution of demographic variables for the Japanese and Chinese samples.

Variable	Category	JAPAN *Percent(%)*	CHINA *Percent(%)*
Age (years old)	20–29	3.2	47.16
	30–39	12.6	38.63
	40–49	22.6	11.14
	50–59	31.8	2.61
	60–69	21.60	0.47
	70–75	8.25	0.00
Personal Income (Unit:yuan/yen)	less than 20 thousand/2 million	28.40	5.69
	20–50 thousand/2–3 million	10.68	8.77
	51–100 thousand/3–4 million	12.14	26.30
	101–150 thousand/4–5 million	10.44	23.93
	151–200 thousand/5–6 million	7.77	15.40
	201–250 thousand/6–7 million	8.25	6.40
	251–300 thousand/7–8 million	5.58	7.35
	301–400 thousand/8–9 million	3.64	2.84
	401–500 thousand/9–10 million	3.64	1.42
	over 500 thousand/ over 10 million	9.47	1.90
Gender	Male	46.36	45.73
	Female	53.64	54.27
Education Level	Junior high school and below	0.73	1.42
	High school	21.84	2.37
	Junior college/Vocational school	21.60	12.56
	University	50.49	74.64
	Graduate school	5.34	9.00
Children Under 18	No	79.85	23.93
	Yes	20.15	76.07
*Obs*		*412*	*422*

Table 4 outlines the measurements related to organic produce awareness through BC. Variables are measured with a five-point Likert scale and further divided into subvariables for Organic (O) and Semi-organic (S) produce.

**Table 4:** Measurement of brand commitment to organic produce.

Variable Name	Describe *(1–5 levels Likert scale)*	*Japan* *Mean (S.D)*	*China* *Mean (S.D)*
*KNOW_O/S*			
	How much do you know about the concept of organic produce?	3.88 (0.73)	3.67 (0.75)
	How much do you know about the concept of semi-organic produce?	2.17 (1.09)	3.98 (0.73)
*BUYEXP_O/S*			
	Have you bought organic produce?	3.01 (1.09)	3.62 (0.84)
	Have you bought semi-organic produce?	1.72 (1.03)	3.85 (0.94)
*TRUST_O/S*	How much do you trust the concept of organic produce?	3.35 (0.74)	3.82 (0.89)
	How much do you trust the concept of semi-organic produce?	3.28 (0.76)	3.96 (0.90)
*SHOP_O/S*			
	There are stores near me that sell organic produce.	2.56 (0.79)	3.36 (0.95)
	Some stores near me sell semi-organic produce.	1.79 (0.90)	3.41 (0.97)
*MEDIA_O/S*			
	I see much information on social media about organic produce.	2.66 (0.92)	3.69 (0.99)
	I see much information on social media about semi-organic produce.	1.83 (0.91)	3.68 (1.05)
*FRIEND_O/S*			
	My family and friends regularly buy organic produce.	2.57 (0.88)	3.56 (0.97)
	My family and friends regularly buy semi-organic produce.	1.80 (0.90)	3.70 (0.96)
*ONLINE_O/S*			
	I know organic produce can be ordered online.	2.87 (1.04)	3.59 (1.02)
	I know semi-organic produce can be ordered online.	1.93 (1.08)	3.71 (1.08)
*Observation*		*412*	*422*

## Results

We analyzed the validity and reliability of the BC for both Japanese and Chinese samples. For the Japanese sample, the Kaiser-Meyer-Olkin (KMO) measure of sampling adequacy and Bartlett’s test of sphericity yielded values of 0.907, and the Cronbach’s α for internal consistency was 0.920. For the Chinese sample, the KMO and Bartlett’s values were 0.789, with a Cronbach’s α of 0.805. These results suggest that the configuration of questionnaire options is valid and reliable. The values in the correlation matrices are shown in S2 Appendix in S1 File.

### Latent profile analysis

Table 5 presents the results of fit indices tests from 2 to 7 profiles. The data analysis was conducted using MPLUS 8.1, adhering to the guidelines provided by Muthén and Muthén [97]. To identify the optimal number of latent classes, we used the TECH11 (the Lo–Mendell–Rubin adjusted likelihood ratio test, LMR-LRT) and TECH14 (the parametric bootstrap likelihood ratio test, BLRT) procedures [98]. We refrained from relying solely on the Akaike information criterion, as it could have inaccurately identified latent classes in larger samples [99]. Instead, the bootstrap likelihood ratio test and BIC were utilized to establish the number of classes [100,101].

**Table 5:** Fit indices for models using LPA.

Profile	AIC	BIC	aBIC	Loglikelihood	Entropy	LMR	BLRT
*JAPAN*							
3	12440.45	12673.67	12489.62	−6162.22	0.96	0.00	0.00
4	12225.07	12518.60	12286.96	−6039.53	0.89	0.01	0.00
5	11977.67	12331.52	12052.28	−5900.84	0.91	0.02	0.00
6	11839.82	12253.99	11927.15	−5816.91	0.92	0.09	0.00
7	11804.26	12278.75	11904.31	−5784.13	0.91	0.89	0.00
*Obs*	*412*						
*CHINA*							
2	15229.27	15403.20	15266.75	−7571.63	0.82	0.00	0.00
3	15069.64	15304.25	15120.19	−7476.82	0.80	0.07	0.00
4	14937.36	15232.64	15000.99	−7395.68	0.82	0.48	0.00
5	14846.30	15202.26	14923.00	−7335.15	0.83	0.06	0.00
6	14817.40	15234.04	14907.19	−7305.70	0.84	0.76	0.00
*Obs*	*422*						

Note: AIC = Akaike information criterion; BIC = Bayesian information criterion; aBIC = adjusted BIC; LMR = Vuong-Lo-Mendell-Rubin likelihood ratio test; BLR = parametric bootstrapped likelihood ratio test.

Based on the BIC and entropy fit evaluations, we segmented consumers from Japan and China into five distinct groups. For the Japanese market, despite profile 6 having a smaller BIC, the LMR was greater than 0.05, leading us to settle on five profiles. The five segments were given the following labels: “Don’t Know” (NO); “Knowledgeable and Willing to Purchase” (TRY); “In Possession of Brand Information and Trusting” (TRU); “High Brand Commitment” (BRA); and “Highly Familiar” (FAM).

**Fig 4:**
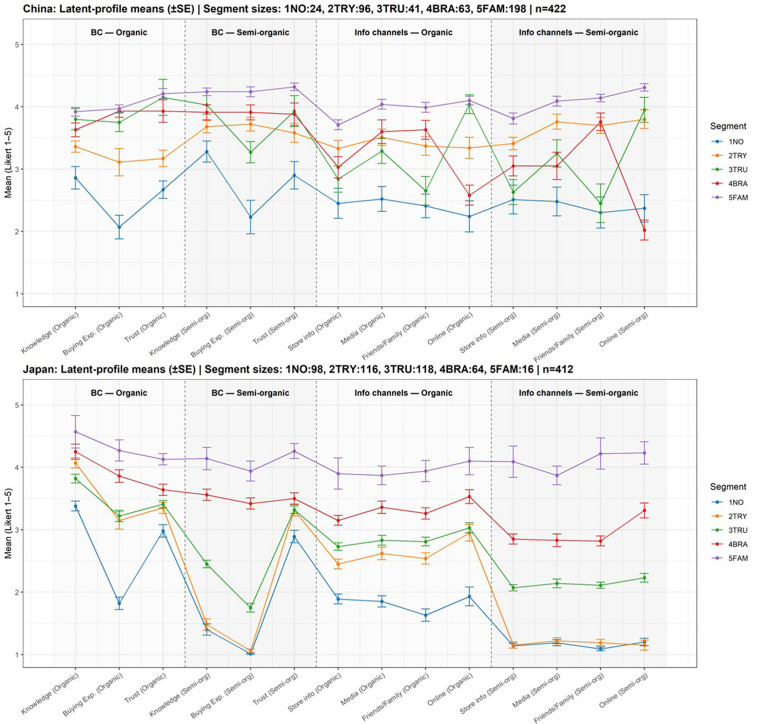
Profile means (±SE) by segment and indicator.

Fig 4 presents the mean scores of various variables across the five profiles, highlighting the specific characteristics of each consumer segment. Results from the ANOVA analysis indicated that the mean differences between different latent classes were statistically significant at the p < 0.05 level.

A notable cross-national difference lies in the profile of the Low-Commitment ‘1NO’ segment. In Japan, this group’s scores for semi-organic products—particularly regarding buying experience and trust—are exceptionally low, approaching the scale minimum. In China, the scores for the ‘1NO’ segment, while still the lowest, are comparatively more moderate. This suggests that Japan’s least engaged consumers have a particularly strong lack of experience with and trust in semi-organic options. Furthermore, Chinese consumers across all segments reported a significantly stronger reliance on online platforms for information about organic products compared to their Japanese counterparts.

### Multiple logistic regression analysis for segments

Subsequently, we treated personal attributes as 0–1 dummy predictor variables and conducted multiple logistic regressions, considering each potential subgroup as independent variables.

Tables 6 and 7 indicate that higher income promotes consumers’ brand commitment of such organic and semi-organic produce in both Japan and China, while an increase in age tends to have the opposite effect. Notably, in China, we observed that families with children are more likely to be familiar with organic produce and are classified into the “Highly Familiar” group.

**Table 6:** Multiple logistic regression analysis for Japanese consumer segments.

*Predict variables*	*TRY*		*TRU*		*BRA*		*FAM*	
	*b*	*OR*	*b*	*OR*	*b*	*OR*	*b*	*OR*
AGE(20 ~ 29 = 0)								
30 ~ 39	−0.54	0.59	−0.05	0.95	−2.06	0.13	−2.03	0.13
40 ~ 49	−0.64	0.53	−0.72	0.49	−1.99	0.14	−2.57*	0.08
50 ~ 59	−0.94	0.39	−0.76	0.47	−2.57**	0.08	−3.26**	0.04
60 ~ 69	−0.21	0.81	−0.37	0.69	−2.53*	0.08	−2.96*	0.05
70 ~ 75	0.95	2.58	0.38	1.46	−1.86	0.16	−1.66	0.19
GENDER	0.31	1.37	0.16	1.18	0.31	1.36	0.28	1.32
INCOME (0 ~ 3 million = 0)								
3 ~ 5 million	0.48	1.61	0.26	1.30	0.86*	2.37	1.57	4.79
over 5 million	0.81*	2.26	1.16***	3.18	1.03**	2.79	2.59**	13.34
EDUC (≥ college = 1)	−0.02	0.98	−0.57*	0.57	0.52	1.69	0.25	1.29
CHILD	0.14	1.15	0.24	1.27	0.61	1.85	−0.30	0.74

Note: *p < .10, **p < .05, ***p < .01. Parameterization using Reference Class 1. *b* = log-odds coefficient from a binary logistic regression; OR=exp(b(odds ratio). OR >1 indicates higher odds of being in the listed segment relative to the reference segment NO.

**Table 7:** Multiple logistic regression analysis for Chinese consumer segments.

*Predict variables*	*TRY*		*TRU*		*BRA*		*FAM*	
	*b*	*OR*	*b*	*OR*	*b*	*OR*	*b*	*OR*
AGE(20 ~ 29 = 0)								
30 ~ 39	−0.37	0.69	−0.91	0.40	−0.84	0.43	−0.50	0.61
40 ~ 49	−1.18	0.31	−2.22*	0.11	−1.85*	0.16	−1.70*	0.19
50 ~ 59	−0.17	0.85	−18.09***	0.00	−1.07	0.34	−2.11	0.12
60 ~ 69	−23.63***	0.00	−22.84***	0.00	−25.24***	0.00	−3.47**	0.03
GENDER	−0.32	0.73	0.09	1.09	0.17	1.18	−0.52	0.60
INCOME (0 ~ 100 thousand = 0)								
100 ~ 200 thousand	0.58	1.78	1.28	3.59	0.86	2.36	0.92	2.50
over 200 thousand	19.75***	–	20.83***	–	20.22***	–	20.23***	–
EDUC (≥ college = 1)	0.21	1.23	1.32	3.75	0.14	1.15	0.93	2.53
CHILD	0.65	1.92	1.28	3.60	1.13	3.09	1.96**	7.08

Note: *p < .10, **p < .05, ***p < .01. Parameterization using Reference Class 1. *b* = log-odds coefficient from a binary logistic regression; OR=exp(b(odds ratio). OR >1 indicates higher odds of being in the listed segment relative to the reference segment NO.

### MWTP for consumer segments

We further investigated the influence of latent segments and external information on consumers’ MWTP. We performed intergroup variability analyses with the “EI-Control” (the group without external information as shown in Table 1) serving as the reference group for both organic (ORGA) and semi-organic (SEMI) produce in comparison to conventional alternatives. Tables 8 and 9 present the results for the Japanese and Chinese markets, respectively.

**Table 8:** Intergroup variability analysis with EI-control as the base group, Japan.

	*CATEGORY*					
	*CABBAGE*		*TOMATO*		*CARROT*	
		*MWTP*		*MWTP*		*MWTP*
ASC	6.98***		6.61***		5.61***	
PRICE	−0.03***		−0.02***		−0.02***	
ORGA	0.79***	26.33	0.29**	14.50	0.61***	30.50
SEMI	0.96***	32.00	0.94***	47.00	0.83***	41.50
ORGA*EI-O	0.20	6.67	0.65***	32.50	0.54***	27.00
ORGA*EI-S	−0.31*	−10.33	0.23	11.50	0.18	9.00
ORGA*EI-OS	−0.16	−5.33	0.08	4.00	0.15	7.50
SEMI*EI-O	0.13	4.33	0.16	8.00	0.56***	28.00
SEMI*EI-S	−0.03	−1.00	0.69***	34.50	0.26	13.00
SEMI*EI-OS	−0.12	−4.00	−0.06	−3.00	0.01	0.50
TRY*ORGA	0.16	5.33	0.12	6.00	0.03	1.50
TRU*ORGA	0.67***	22.33	0.52***	26.00	0.26	13.00
BRA*ORGA	0.86***	28.67	0.98***	49.00	0.64***	32.00
FAM*ORGA	1.58***	52.67	1.32***	66.00	1.31***	65.50
TRY*SEMI	0.07	2.33	−0.52**	−26.00	−0.06	−3.00
TRU*SEMI	0.61***	20.33	−0.22	−11.00	0.28*	14.00
BRA*SEMI	0.93***	31.00	0.08	4.00	0.73***	36.50
FAM*SEMI	2.18***	72.67	−0.86**	−43.00	1.20***	60.00
*R2*	0.384		0.388		0.308	
*Adj- R2*	0.379		0.383		0.303	
*LR-test*	2779***		2813***		2230***	
*Obs*	*412*					

Note: *p < .10, **p < .05, ***p < .01.

**Table 9:** Intergroup variability analysis with EI-control as the base group, China.

	*CATEGORY*					
	*CABBAGE*		*TOMATO*		*CARROT*	
		*MWTP*		*MWTP*		*MWTP*
ASC	1.85***		2.33***		1.55***	
PRICE	−0.14***		−0.15***		−0.14***	
ORGA	0.85***	6.07	0.70***	4.67	1.44***	10.29
SEMI	1.31***	9.36	0.50**	3.33	1.56***	11.14
ORGA*EI-O	0.42***	3.00	0.22*	1.47	0.20	1.43
ORGA*EI-S	−0.20	−1.43	−0.32**	−2.13	−0.20	−1.43
ORGA*EI-OS	0.16	1.14	0.01	0.07	0.06	0.43
SEMI*EI-O	0.14	1.00	0.11	0.73	0.09	0.64
SEMI*EI-S	0.07	0.50	0.05	0.33	−0.07	−0.50
SEMI*EI-OS	0.11	0.79	0.02	0.13	0.06	0.43
TRY*ORGA	0.21	1.50	0.46*	3.07	−0.04	−0.29
TRU*ORGA	0.29	2.07	0.42	2.80	0.09	0.64
BRA*ORGA	0.67***	4.79	0.98***	6.53	0.21	1.50
FAM*ORGA	0.42*	3.00	0.68***	4.53	0.09	0.64
TRY*SEMI	0.16	1.14	0.42	2.80	0.02	0.14
TRU*SEMI	0.39	2.79	0.26	1.73	0.03	0.21
BRA*SEMI	0.61*	4.36	0.78***	5.20	0.35	2.50
FAM*SEMI	0.34	2.43	0.50**	3.33	0.05	0.36
*R2*	0.216		0.260		0.255	
*Adj- R2*	0.211		0.255		0.251	
*LR-test*	1600***		1931***		1895***	
*Obs*	*422*					

Note: *p < .10, **p < .05, ***p < .01.

For the Japanese respondents, latent segments with elevated brand commitment exhibited a heightened MWTP, supporting H1a (High brand commitment increases MWTP for organic produce), indicating that consumers with high brand commitment exhibit a greater MWTP for organic produce. Significant positive effects were observed across all three organic product categories (cabbage, tomatoes, and carrots), with MWTP particularly high for carrots and tomatoes. H1b, while validated for cabbage and carrots in the BRA*SEMI variable, was not supported for tomatoes in TRY*SEMI. The interaction terms ORGA*EI-O demonstrated significant positive effects on MWTP for tomatoes and carrots, supporting H2a (external information about organic produce increases MWTP for organic produce), though this was not the case for cabbage. For semi-organic produce, SEMI*EI-O showed a significant effect for carrots but not for cabbage or tomatoes, providing partial support for H2b.

Additionally, the latent profile analysis of brand commitment revealed distinct patterns in MWTP across different consumer segments. The “Highly Familiar” segment consistently exhibited the highest MWTP for both organic and semi-organic produce, indicating strong brand commitment and a higher willingness to pay. Conversely, the “Knowledgeable and Willing to Purchase” segment displayed a more nuanced response, particularly for semi-organic tomatoes, where their MWTP was lower compared to other products. This suggests that consumer segments defined by varying levels of brand commitment respond differently to organic and semi-organic products, further highlighting the role of external information in shaping MWTP behavior.

For the Chinese respondents, neither H1a nor H1b were supported in the carrot category. However, for cabbages, segments with higher BC showed an increased MWTP, corroborating H1a and H1b except FAM*SEMI segment. Specifically, in the tomato category, segments with pronounced brand commitment had a higher MWTP, aligning with H1a and H1b. Moreover, consumers in the “High Brand Commitment” segment already have a high MWTP and are willing to pay more for organic cabbage compared to organic tomatoes. External information supported H2a for both cabbages and tomatoes from ORGA*EI-O for tomatoes and cabbages, but all categories refuted H2b. The interaction terms ORGA*EI-O displayed a pattern like that observed in Japan for tomatoes, though there were notable differences in significance for cabbages and carrots. This suggests that external information has a significant positive effect on MWTP when consumers are provided with the definition of organic tomatoes.

Joint Wald tests reject equality of the price and semi-organic attribute coefficients between China and Japan for all three products (Cabbage: χ² = 68.58, df = 3, p < 0.001; Tomato: χ² = 115.46, df = 3, p < 0.001; Carrot: χ² = 174.37, df = 3, p < 0.001), indicating materially different taste parameters across markets (S3–S5 Appendix in S1 File). Also, Cross-country differences in external information effects are uniformly insignificant for all three products.

## Discussion

First, brand commitment helps delineate Chinese and Japanese consumers’ perceptions of organic food but also maps directly onto segment-specific marketing levers [14,36,37]. Japanese consumers are less familiar with semi-organic produce. This lack of familiarity can be attributed to the informal labeling practices in Japan, where these products are often referred to as “pesticide-reduced” or “pesticide-free.” The absence of a uniform labeling or brand name impedes consumers’ abilities to access accurate information about semi-organic produce [3,102]. Conversely, in China, semi-organic produce (or green food) is distinctly identified with a consistent brand name and certification mark, facilitating clearer consumer comprehension and heightened brand recognition. For low- brand commitment consumers, the organic price premium is the primary deterrent; lowering perceived risk via entry-level packs, targeted discounts, and simple, high-salience cues are more effective than additional definitions. For higher brand commitment segments, messages should shift from definitions to specific depth (certifying body, inspection dates, traceability) and attribute clarity, defending price premium by reinforcing long-term memory in consumers’ choices.

Second, WTP across brand commitment segments indicates notable distinctions between Chinese and Japanese consumers, as evident by the marked preference for organic attributes among the “High Brand Commitment” and “Highly Familiar” segments in both countries. For instance, they perceive semi-organic tomatoes differently. Even highly familiar Japanese consumers are less inclined towards this product, contrary to their Chinese counterparts. A significant finding is that while Japanese consumers’ WTP increases with higher latent categories, Chinese consumers in the “High Brand Commitment” exhibit a higher WTP than those in “Highly Familiar”. We believe that there are more middle-class individuals in “High Brand Commitment” in China, who are the main consumers of organic food. This also corroborates the findings of Yuan et al. [76], who indicated that consumers’ environmental awareness plays a more critical role in the consumption of organic products than external information. It is noteworthy that the proportion of respondents in “Highly Familiar” is relatively high, suggesting that the accuracy of the brand commitment measurement still needs improvement. Furthermore, Chinese consumers do not display the same enthusiasm for carrots as seen in Japan, which we speculate might be due to the absence of popular health beverages centered around organic carrots in China, a trend more prevalent in Japan [103,104].

Third, concerning external information, the ramifications of information related to organic agricultural products can be observed in both nations. This revelation carries practical implications. Educating consumers, especially those with limited insight into organic agriculture, about stringent production processes, certification protocols, and the inherent value of organic farming is crucial to bolster their purchasing intent and brand consciousness. For both organic and semi-organic attributes, consumers’ reactions diverged across produce types. Organic external information significantly boosts the WTP for tomatoes, highlighting tomatoes’ potential as a favored organic product across regions [105,106]. Yet, for the semi-organic external information, neither Chinese nor Japanese consumers exhibited a marked preference for carrots over tomatoes and cabbage. Both organic and semi-organic external information, exposed to a dual definition, did not diverge significantly from the control group. This outcome implies that the EI-OS participants do not harbor a pronounced bias towards either information type. Particularly in China, the external information for carrots is statistically null (Table 9). First, external information emphasized certification definitions rather than sensory/culinary cues; for carrots, WTP may be driven more by perceived sweetness/crunch and household use-cases, so definition heavy external information does not shift utility. Second, category risk salience: Chinese consumers may perceive pesticide risk as more acute for leafy vegetables than for root vegetables; if baseline risk is seen as lower for carrots, certification messages yield smaller trust gains relative to tomatoes or cabbage. Thus, it is important to craft distinct messaging and branding to distinguish semi-organic farming approaches from traditional organic agriculture to enhance their market positioning in both China and Japan.

Nevertheless, some limitations must be acknowledged. Latent profile analysis omitted factors such as “environmental protection awareness” and “ideology,” which have been underscored in previous research [33,34,107]. Given the unique political landscapes of China and Japan, national environmental protocols and ideological stances exert considerable influence. Additionally, our study did not prioritize attribute non-attendance [108,109]. The online survey methodology also presented challenges in controlling individual characteristics, indicating the potential need for in-depth studies on targeted demographics, such as college graduates or homemakers [110]. Finally, expanding the sample size to include and compare consumer preferences for organic products between urban and rural areas remains an unexplored area.

These findings provide critical insights into the intricate relationship between external information and consumers’ WTP for produce alternatives. Further, they underscore the significant role of market-specific characteristics in shaping consumers’ preferences and purchase decisions. It is essential to carefully analyze latent segments of consumers before developing advertisements and providing information according to different consumer categories to enhance their purchase intentions. In addition to cost reduction, focusing on customized marketing strategies for high-income groups, which strongly prefer organic agricultural products, will help increase profits.

## Conclusions

Our research findings from both the Japanese and Chinese markets underline the importance of disseminating knowledge and understanding consumer segments in marketing organic produce based on multiple-store memory model. Our study adds to the existing literature by highlighting the nuanced differences in consumer preferences between urban areas in Japan and China, emphasizing the role of brand commitment (LTM) and external information (STM). Our findings underscore that the effectiveness of external information is highly dependent on consumers’ pre-existing cognitive structures and the specific market context.

From a policy perspective, our results yield two primary implications. First, the structural design of a certification system is a critical policy instrument. The two markets demonstrate distinct approaches: China’s “Green Food” program functions as a centralized, logo-based brand, which appears to facilitate the construction of a consistent long-term memory schema. In contrast, Japan’s official ‘Specially Cultivated’ guidelines rely on precise, text-based descriptions rather than a singular visual brand. This guideline-based approach, while transparent, may require greater cognitive effort from consumers, potentially leading to the less unified brand recognition observed in our findings. Second, our results highlight the limitations of uniform communication strategies. Public information campaigns should be tailored to consumers’ varying levels of brand commitment. Interventions should differentiate their messaging—using simple, high-salience, trust-building cues for segments with undeveloped long-term memory, while providing detailed, reinforcing data to defend price premiums for segments with well-established long-term memory.

Considering the increasing global focus on sustainable consumption and health, further research in this area can help stakeholders understand the key drivers and barriers to the adoption of organic and semi-organic products in different cultural contexts. These insights can guide the development of effective strategies to promote sustainable food consumption, contributing to the broader global goals of health and sustainability.

## Supporting information

S1 File S1 Appendix. Major cities (provinces or Ken) for selection of survey respondents in Japan and China. S2 Appendix. Correlation matrix of BC and BI variables in the Japanese and Chinese sample. Correlation matrix of variables in the Japanese sample. S3 Appendix. Cross-country Wald tests. S4 Appendix. Within-country Wald tests for EI effects. S5 Appendix. Cross-country Wald tests for EI effects.
(DOCX)
