# Supplementary material for: The role of brand commitment and external information in urban consumers’ organic produce choices: Evidence from Japan and China
Source: PLoS One. 2025 Nov 21;20(11):e0337225. doi: 10.1371/journal.pone.0337225 (PMC12637908; doi:10.1371/journal.pone.0337225)
Supplement: S1 File — S1 Appendix. Major cities (provinces or Ken) for selection of survey respondents in Japan and China. S2 Appendix. Correlation matrix of BC and BI variables in the Japanese and Chinese sample. Correlation matrix of variables in the Japanese sample. S3 Appendix. Cross-country Wald tests. S4 Appendix. Within-country Wald tests for EI effects. S5 Appendix. Cross-country Wald tests for EI effects. (DOCX) [file pone.0337225.s001.docx]

**Appendix**

## S1 Appendix. Major cities (provinces or Ken) for selection of survey respondents in Japan and China.

| **Japan** | **China** |
| --- | --- |
| Tokyo, | Beijing, Shanghai, |
| Hokkaido, Miyagi, Chiba, Kanagawa, Saitama, Aichi, Osaka, Fukuoka | Tianjin, Jiangsu, Zhejiang, Anhui, Fujian, Shandong, Henan, Hubei, Hunan, Guangdong, Chongqing, Sichuan, Yunnan, Shanxi |

Note: In Japan, the data represent responses from these prefectures, which include government-designated cities, but do not specify which city within the prefecture the respondents are from.

## S2 Appendix. Correlation matrix of BC and BI variables in the Japanese and Chinese sample.Correlation matrix of variables in the Japanese sample.

|  | KNOW_O | BUYEXP_O | TRUST_O | SHOP_O | MEDIA_O | FRIEND_O | ONLINE_O | KNOW_S | BUYEXP_S | TRUST_S | SHOP_S | MEDIA_S | FRIEND_S | ONLINE_S |
| --- | --- | --- | --- | --- | --- | --- | --- | --- | --- | --- | --- | --- | --- | --- |
| KNOW_O | 1 |  |  |  |  |  |  |  |  |  |  |  |  |  |
| BUYEXP_O | 0.542 | 1 |  |  |  |  |  |  |  |  |  |  |  |  |
| TRUST_O | 0.226 | 0.346 | 1 |  |  |  |  |  |  |  |  |  |  |  |
| SHOP_O | 0.304 | 0.519 | 0.306 | 1 |  |  |  |  |  |  |  |  |  |  |
| MEDIA_O | 0.345 | 0.536 | 0.264 | 0.497 | 1 |  |  |  |  |  |  |  |  |  |
| FRIEND_O | 0.393 | 0.632 | 0.347 | 0.542 | 0.557 | 1 |  |  |  |  |  |  |  |  |
| ONLINE_O | 0.479 | 0.485 | 0.134 | 0.378 | 0.442 | 0.452 | 1 |  |  |  |  |  |  |  |
| KNOW_S | 0.298 | 0.452 | 0.275 | 0.438 | 0.419 | 0.462 | 0.436 | 1 |  |  |  |  |  |  |
| BUYEXP_S | 0.273 | 0.489 | 0.284 | 0.482 | 0.432 | 0.518 | 0.361 | 0.742 | 1 |  |  |  |  |  |
| TRUST_S | 0.22 | 0.336 | 0.657 | 0.221 | 0.24 | 0.305 | 0.145 | 0.272 | 0.264 | 1 |  |  |  |  |
| SHOP_S | 0.194 | 0.431 | 0.253 | 0.564 | 0.433 | 0.527 | 0.329 | 0.686 | 0.778 | 0.261 | 1 |  |  |  |
| MEDIA_S | 0.244 | 0.424 | 0.258 | 0.462 | 0.575 | 0.499 | 0.342 | 0.676 | 0.7 | 0.246 | 0.739 | 1 |  |  |
| FRIEND_S | 0.225 | 0.454 | 0.314 | 0.516 | 0.496 | 0.627 | 0.387 | 0.681 | 0.776 | 0.293 | 0.796 | 0.781 | 1 |  |
| ONLINE_S | 0.282 | 0.429 | 0.231 | 0.44 | 0.422 | 0.437 | 0.499 | 0.761 | 0.746 | 0.277 | 0.724 | 0.734 | 0.725 | 1 |

**Correlation matrix of variables in the Chinese sample**

|  | KNOW_O | BUYEXP_O | TRUST_O | SHOP_O | MEDIA_O | FRIEND_O | ONLINE_O | KNOW_S | BUYEXP_S | TRUST_S | SHOP_S | MEDIA_S | FRIEND_S | ONLINE_S |
| --- | --- | --- | --- | --- | --- | --- | --- | --- | --- | --- | --- | --- | --- | --- |
| KNOW_O | 1 |  |  |  |  |  |  |  |  |  |  |  |  |  |
| BUYEXP_O | 0.304 | 1 |  |  |  |  |  |  |  |  |  |  |  |  |
| TRUST_O | 0.325 | 0.38 | 1 |  |  |  |  |  |  |  |  |  |  |  |
| SHOP_O | 0.2 | 0.246 | 0.143 | 1 |  |  |  |  |  |  |  |  |  |  |
| MEDIA_O | 0.138 | 0.208 | 0.192 | 0.205 | 1 |  |  |  |  |  |  |  |  |  |
| FRIEND_O | 0.196 | 0.291 | 0.242 | 0.245 | 0.289 | 1 |  |  |  |  |  |  |  |  |
| ONLINE_O | 0.278 | 0.282 | 0.314 | 0.209 | 0.148 | 0.199 | 1 |  |  |  |  |  |  |  |
| KNOW_S | 0.372 | 0.21 | 0.171 | 0.113 | 0.17 | 0.133 | 0.198 | 1 |  |  |  |  |  |  |
| BUYEXP_S | 0.143 | 0.315 | 0.276 | 0.15 | 0.253 | 0.307 | 0.159 | 0.212 | 1 |  |  |  |  |  |
| TRUST_S | 0.089 | 0.181 | 0.425 | 0.15 | 0.194 | 0.2 | 0.22 | 0.283 | 0.24 | 1 |  |  |  |  |
| SHOP_S | 0.156 | 0.141 | 0.087 | 0.405 | 0.272 | 0.213 | 0.193 | 0.257 | 0.25 | 0.168 | 1 |  |  |  |
| MEDIA_S | 0.152 | 0.196 | 0.065 | 0.296 | 0.376 | 0.234 | 0.288 | 0.197 | 0.304 | 0.104 | 0.273 | 1 |  |  |
| FRIEND_S | 0.098 | 0.225 | 0.166 | 0.201 | 0.282 | 0.446 | 0.111 | 0.168 | 0.401 | 0.281 | 0.313 | 0.26 | 1 |  |
| ONLINE_S | 0.172 | 0.132 | 0.18 | 0.226 | 0.195 | 0.131 | 0.529 | 0.214 | 0.195 | 0.227 | 0.269 | 0.349 | 0.217 | 1 |

## S3 Appendix. Cross-country Wald tests

| vegetable | Chi-square | df | p-value | sig |
| --- | --- | --- | --- | --- |
| Cabbage | 68.57936 | 3 | 0.000 | *** |
| Tomato | 115.45679 | 3 | 0.000 | *** |
| Carrot | 174.36603 | 3 | 0.000 | *** |

## S4 Appendix. Within-country Wald tests for EI effects

| vegetable | Side | Info | test | Chi-square | df | p-value |
| --- | --- | --- | --- | --- | --- | --- |
| Cabbage | JP | EIO | ORGANIC:EIO = 0, SPEC:EIO = 0 | 1.626 | 2 | 0.444 |
| Cabbage | JP | EIS | ORGANIC:EIS = 0, SPEC:EIS = 0 | 3.562 | 2 | 0.168 |
| Cabbage | JP | EIOS | ORGANIC:EIOS = 0, SPEC:EIOS = 0 | 4.119 | 2 | 0.128 |
| Cabbage | CN | EIO | ORGANIC:EIO = 0, SPEC:EIO = 0, ORGANIC:EIO:CHINA = 0, SPEC:EIO:CHINA = 0 | 3.536 | 4 | 0.472 |
| Cabbage | CN | EIS | ORGANIC:EIS = 0, SPEC:EIS = 0, ORGANIC:EIS:CHINA = 0, SPEC:EIS:CHINA = 0 | 5.017 | 4 | 0.286 |
| Cabbage | CN | EIOS | ORGANIC:EIOS = 0, SPEC:EIOS = 0, ORGANIC:EIOS:CHINA = 0, SPEC:EIOS:CHINA = 0 | 6.254 | 4 | 0.181 |
| Tomato | JP | EIO | ORGANIC:EIO = 0, SPEC:EIO = 0 | 1.226 | 2 | 0.542 |
| Tomato | JP | EIS | ORGANIC:EIS = 0, SPEC:EIS = 0 | 0.220 | 2 | 0.896 |
| Tomato | JP | EIOS | ORGANIC:EIOS = 0, SPEC:EIOS = 0 | 2.935 | 2 | 0.231 |
| Tomato | CN | EIO | ORGANIC:EIO = 0, SPEC:EIO = 0, ORGANIC:EIO:CHINA = 0, SPEC:EIO:CHINA = 0 | 1.990 | 4 | 0.738 |
| Tomato | CN | EIS | ORGANIC:EIS = 0, SPEC:EIS = 0, ORGANIC:EIS:CHINA = 0, SPEC:EIS:CHINA = 0 | 2.232 | 4 | 0.693 |
| Tomato | CN | EIOS | ORGANIC:EIOS = 0, SPEC:EIOS = 0, ORGANIC:EIOS:CHINA = 0, SPEC:EIOS:CHINA = 0 | 4.632 | 4 | 0.327 |
| Carrot | JP | EIO | ORGANIC:EIO = 0, SPEC:EIO = 0 | 5.973 | 2 | 0.050 |
| Carrot | JP | EIS | ORGANIC:EIS = 0, SPEC:EIS = 0 | 2.004 | 2 | 0.367 |
| Carrot | JP | EIOS | ORGANIC:EIOS = 0, SPEC:EIOS = 0 | 1.252 | 2 | 0.535 |
| Carrot | CN | EIO | ORGANIC:EIO = 0, SPEC:EIO = 0, ORGANIC:EIO:CHINA = 0, SPEC:EIO:CHINA = 0 | 7.621 | 4 | 0.106 |
| Carrot | CN | EIS | ORGANIC:EIS = 0, SPEC:EIS = 0, ORGANIC:EIS:CHINA = 0, SPEC:EIS:CHINA = 0 | 4.778 | 4 | 0.311 |
| Carrot | CN | EIOS | ORGANIC:EIOS = 0, SPEC:EIOS = 0, ORGANIC:EIOS:CHINA = 0, SPEC:EIOS:CHINA = 0 | 1.415 | 4 | 0.842 |

## S5 Appendix. Cross-country Wald tests for EI effects

| vegetable | Info | test | Chi-square | df | p-value |
| --- | --- | --- | --- | --- | --- |
| Cabbage | EIO | ORGANIC:EIO:CHINA = 0, SPEC:EIO:CHINA = 0 | 0.630 | 2 | 0.730 |
| Cabbage | EIS | ORGANIC:EIS:CHINA = 0, SPEC:EIS:CHINA = 0 | 0.646 | 2 | 0.724 |
| Cabbage | EIOS | ORGANIC:EIOS:CHINA = 0, SPEC:EIOS:CHINA = 0 | 0.821 | 2 | 0.663 |
| Tomato | EIO | ORGANIC:EIO:CHINA = 0, SPEC:EIO:CHINA = 0 | 1.885 | 2 | 0.390 |
| Tomato | EIS | ORGANIC:EIS:CHINA = 0, SPEC:EIS:CHINA = 0 | 1.213 | 2 | 0.545 |
| Tomato | EIOS | ORGANIC:EIOS:CHINA = 0, SPEC:EIOS:CHINA = 0 | 1.745 | 2 | 0.418 |
| Carrot | EIO | ORGANIC:EIO:CHINA = 0, SPEC:EIO:CHINA = 0 | 5.147 | 2 | 0.076 |
| Carrot | EIS | ORGANIC:EIS:CHINA = 0, SPEC:EIS:CHINA = 0 | 3.525 | 2 | 0.172 |
| Carrot | EIOS | ORGANIC:EIOS:CHINA = 0, SPEC:EIOS:CHINA = 0 | 1.303 | 2 | 0.521 |

Notes: Wald tests use cluster-robust (ID) variance from conditional logit; significance codes: *** p<0.001, ** p<0.01, * p<0.05.
